# HIV-1 Tat Length: Comparative and Functional Considerations

**DOI:** 10.3389/fmicb.2020.00444

**Published:** 2020-03-24

**Authors:** Anthony R. Mele, Jamie Marino, Will Dampier, Brian Wigdahl, Michael R. Nonnemacher

**Affiliations:** ^1^Department of Microbiology and Immunology, Drexel University College of Medicine, Philadelphia, PA, United States; ^2^Center for Molecular Virology and Translational Neuroscience, Institute for Molecular Medicine and Infectious Disease, Drexel University College of Medicine, Philadelphia, PA, United States; ^3^School of Biomedical Engineering, Science and Health Systems, Drexel University, Philadelphia, PA, United States; ^4^Sidney Kimmel Cancer Center, Thomas Jefferson University, Philadelphia, PA, United States

**Keywords:** HIV-1, Tat, genetic variation, transcription, truncation

## Discussion

Human immunodeficiency virus type 1 (HIV-1) has been shown to encode a small basic protein known as the transactivator of transcription (Tat) (Rice and Mathews, 1988) and as recently reviewed (Spector et al., 2019). Tat is a multifunctional protein, though it is primarily responsible for recruitment of the host positive transcription elongation factor b (P-TEFb) by interaction with an RNA stem-loop designated the transactivation response (TAR) element, which is encoded by the viral long terminal repeat (LTR) (Dingwall et al., 1989; Li et al., 2011; Khoury et al., 2018). This interaction leads to efficient transactivation of HIV-1 and this function has been shown to be contained within the first exon of Tat (residues 1–58) (Kuppuswamy et al., 1989; Link et al., 2019). A number of other functional properties have been associated with Tat and many of these activities have been noted once the protein resides in extracellular space (Khan et al., 2019). In this regard, Tat has been shown to be secreted through a number of different mechanisms, as previously reviewed (Mele et al., 2018) which are dependent on residues at positions 11 and 49–51 and their interactions with phosphatidylinositol-4,5-bisphosphate (PtdIns(4,5)P_2_), a lipid in the plasma membrane (Rayne et al., 2010b). Tat secretion is a highly active process, and concentrations of extracellular Tat have been found (200 pg/ml to 6.5 ng/ml) in the cerebral spinal fluid (CSF) of HIV-1-infected patients who are well-suppressed on antiretroviral therapy (Johnson et al., 2013; Henderson et al., 2019). Within the central nervous system (CNS), extracellular Tat can recruit peripheral immune cells that has been shown to lead to low levels of chronic inflammation through activation of bystander cells and release of pro-inflammatory cytokines, such as interleukin (IL) 1 beta (IL-1β), IL-6, and monocyte chemoattractant protein 1 (MCP-1) from monocytes and macrophages (Hofman et al., 1994; Albini et al., 1998; Hudson et al., 2000; Pulliam et al., 2007; Rayne et al., 2010a; Bachani et al., 2013). Additionally, Tat has been shown to be directly neurotoxic through hyper-activation of neurons (Fields et al., 2015), which contributes to the development of HIV-1-associated neurocognitive disorders (HAND) (Gaskill et al., 2017). These functional properties, however, are dependent on the amino acid mutations present at a number of different Tat residues.

HIV-1 is predisposed to genetic variation, which is caused by factors such as the error-prone viral reverse transcriptase in conjunction with an array of selective pressures including the host immune response (Li et al., 2012). Tat, which is subject to genetic variation (Dampier et al., 2016) during the course of HIV disease, may also result in a number of alterations in amino acid residues that have been associated with altered function. One such example of a functional alteration would be a reduction in LTR transactivation (Boven et al., 2007; Ronsard et al., 2017), which can occur with only a single residue mutation, such as position 11 substitutions to either alanine, phenylalanine, or leucine (Yezid et al., 2009), a glutamine substitution at position 50 (Brès et al., 2002), or an alanine substitution at position 51 (Van Duyne et al., 2008). As discussed, HIV-1 Tat has been shown to be encoded by two exons, which after being alternatively spliced and translated, results in a 101 amino acid protein. We (Link et al., 2019), and others (Jeang et al., 1999; Marcello et al., 2001; López-Huertas et al., 2010; van der Kuyl et al., 2018), have demonstrated that the most prevalent Tat length within subtype B HIV-1-infected patient samples has been shown to be Tat 101 (>85%), while other widely used forms of Tat, including Tat 86 and Tat 72, occur at much lower frequencies. It is important to note that Tat 86 is still found in patient sequences albeit at a low occurrence as we have previously shown (Link et al., 2019) (CARES Cohort 15.38/207.01; 7.42% and LANL 58.91/1483; 3.97%) and as reported with the BEEHIVE Cohort (7/291; 2.4%) (van der Kuyl et al., 2018). They have also shown that in an analysis of Tat sequences in LANL from other subtypes that Tat 86 might be more dominant in subtypes D; 43/56 (76%) of subtype D viruses, 3/4 (75%) of subtype H viruses, 22/812 (2.7%) of subtype B viruses, and 3/382 (0.8%) of subtype C viruses; but not in subtypes A (0/130), F (0/32), G (0/6) or J/K (0/15) (van der Kuyl et al., 2018). However, this may be due to the small number of sequences available for each of these subtypes. We observed a similar trend when examining the world-wide distribution of subtype B sequences and found certain countries, such as France, had a reduced number of sequences submitted to LANL, resulting in their most frequent length being Tat 86 (Link et al., 2019). Given this observation, the following discussion has examined functional alterations that have been observed between Tat 86 and Tat 101, primarily from studies using subtype B or C. In order to accurately understand the biological properties that can be influenced by Tat, it would seem important to utilize the most prevalent and biologically relevant protein.

**Figure 1:**
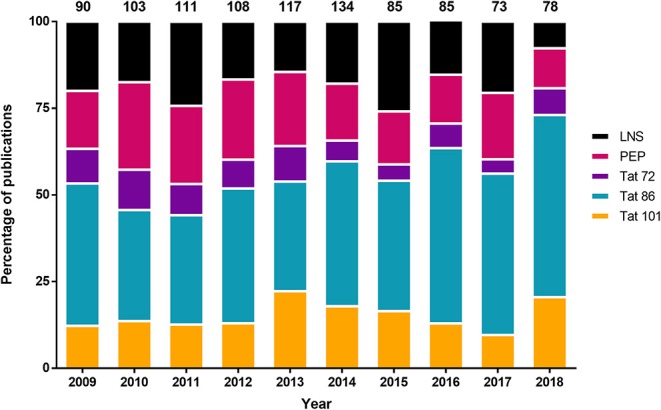
Distribution of the different Tat lengths used in the literature from 2009 to 2018. The 101 residue Tat was not the most frequently utilized Tat length variant in the literature from publicly available publications on PubMed. The parameters used on the PubMed search were: (HIV-1 Tat) NOT “review”[Publication Type], 2009/01/01 to 2018/12/31, and free full text. The number of publications per year were converted into a percentage (y-axis), and the total number of publications was listed above the figure. Refer to Supplemental Table 1 for more information regarding the categories of Tat. Tat length not stated in publication (LNS); Tat peptides (PEP).

It appears that most HIV-1 Tat investigations have utilized Tat 86, however, the accuracy of this statement has not been examined. In order to quantify the frequency of particular Tat truncations in the literature, a meta-analysis of publicly available publications was performed for the last 10 years. A PubMed search was performed using the following parameters: (HIV-1 Tat) NOT “review”[Publication Type], 2009/01/01 to 2018/12/31, and free full text. At the time this manuscript was drafted, there were 973 publications, which were then read and assessed for their utilization of different forms of Tat (Figure 1). There were an additional 407 publications that were not publicly available and thus were not assessed, due to access limitations. The results included expression of Tat within animal models, plasmids, or purified recombinant proteins, as well as molecular simulations and sequencing studies. One of the more prominent uses of Tat was as a fusion peptide for intracellular trafficking of another protein. This was listed as *Tat peptides* (*PEP*), however, this also included a number of additional minor truncations, such as Tat 82 (Dutta and Roy, 2015). Publications that did not explicitly state the length of Tat or the molecular clone it was derived from were listed as *Tat length not stated in publication* (*LNS*) [18.29% (178 of 973)] (Supplemental Table 1). There were a few results that examined SIV rather than HIV, and those were not included in Figure 1. As shown, 40.18% (391 of 973) of the publications used Tat 86, while only 15.51% (151 of 973) used Tat 101. Over each of the 10 years there was an average of 24 more Tat 86 papers relative to Tat 101. A paired Student's *t*-test of the number of publications for Tat 101 compared to Tat 86 each year over this 10-year period was statistically significant (*p* < 0.0001). The results of this meta-analysis supports the statement that Tat 86 is used more frequently than Tat 101 even though it has been encountered much less frequently in HIV-1-infected patient samples. Furthermore, a surprising number of publications did not clearly describe the length of Tat in the study, which may affect our understanding of Tat functionality.

Tat is a multifunctional protein and premature truncations such as Tat 72 or 86 have been demonstrated to alter its functional properties. Studies observed that expression of Tat 101 significantly altered mitochondrial DNA transcription, mitochondrial content, and their distribution within peripheral blood lymphocytes in comparison to Tat 72 (Rodríguez-Mora et al., 2015). Additional studies arrived at a similar conclusion where they observed that Jurkat cells expressing Tat 101 had altered cell morphology, proliferation, chemotaxis, polarization, and actin polymerization, but these effects were not present in cells expressing Tat 72 (López-Huertas et al., 2010). Furthermore, the second exon of Tat has also been demonstrated to be critical in reducing host innate responses, such as downregulating interferon-stimulating genes (Kukkonen et al., 2014). When examining the cellular gene modulation of different Tat lengths in THP-1 cells, a monocytic cell line, and in human primary monocyte-derived macrophages, it was found that an HIV-1_SF2_ molecular clone expressing only the first exon of Tat (Tat 72) significantly increased the RNA and protein of innate response genes, such as Stat-1, MX2, and IRF-7, when compared to Tat 101, which resulted in a downregulation of these genes (Kukkonen et al., 2014). Tat 101 has also been demonstrated to induce ten-fold more IL-2 secretion vs. the amount that Tat 72 was capable of in Jurkat cells (Johnson et al., 2013).

Another example related to the functional properties of the second exon of Tat has been the dependence on the NF-κB-associated motif, ESKKKVE, which localize to residues 86–92 in HIV-1 subtype B Tat and would not be contained in the Tat 72 or Tat 86 versions of the protein. Expression of Tat 101 increased anti-apoptotic proteins, such as BCL2, and was demonstrated to cause a delay in FasL-mediated apoptosis of human peripheral blood lymphocytes as well as Jurkat cells (López-Huertas et al., 2013). This effect was not seen when examining Tat 72, to which the authors concluded was dependent on the NF-κB-associated motif, Tat 86 was not tested. Further characterization of this motif has also demonstrated that the molecular clone HIV-1_89.6_ expressing Tat 72 replicated at significantly lower levels than Tat 101-expressing HIV-1_89.6_ in human primary blood lymphocytes (Mahlknecht et al., 2008). Additional mutations were made within and downstream of the ESKKKVE motif in the Tat 101-expressing virus and also demonstrated the necessity for the glutamic acids (positions 92, 94, and 96) and lysines (positions 88, 89, and 90) in this particular activity. Tat function is commonly assessed via an LTR transactivation assay, but as shown in this publication, LTR transactivation may not be indicative of viral replication if the Tat 101 protein is not used.

These results demonstrate that within the past decade, investigations have favored the use of Tat 86. While this may not alter transactivation function of Tat, particularly within a plasmid expression system (Link et al., 2019), there may be alterations in other mechanisms, as described above. As we have discussed, the biological relevance of Tat length within animal models may be another key distinguishing assay to assess differences in function. Animal models are crucial for understanding Tat, specifically due to Tat being measurably expressed in patients with low-to-undetectable viremia or without detecting other HIV-1 proteins (Johnson et al., 2013; Henderson et al., 2019). Tat animal models often employ direct injection of Tat 72 or 86 into the brain (Aksenov et al., 2001, 2003; Cass et al., 2003; Fitting et al., 2008; Agrawal et al., 2012), however, this is not the only method of Tat exposure. Mice have also been exposed to repeated intranasal administration of Tat 86 and were able to demonstrate trafficking of Tat into the CNS, via the olfactory bulb (Pulliam et al., 2007). However, this model was not examined with Tat 101, and it is unknown if the trafficking patterns would be similar. In order to more closely model neurocognitive impairment within patients, use of a chronic exposure animal model that continuously expresses Tat may be of particular importance in determining the *in vivo* functional properties of Tat 101 vs. other length Tat proteins.

To our knowledge, there are no transgenic animal models that express Tat 101 (Soontornniyomkij et al., 2016; Langford et al., 2018; Green et al., 2019). The iTat model, which expresses Tat in a doxycycline-dependent manner that models chronic exposure, is a widely utilized model of HAND, however, it also does not express the Tat 101 protein (Fan et al., 2016; Langford et al., 2018). Similarly, the rtTA-Tat mouse model also expresses Tat 86 under the control of a glial fibrillary acidic protein (GFAP) promoter, which has resulted in low levels of chronic inflammation (Bruce-Keller et al., 2008; Dickens et al., 2017). These mice were observed for an entire year, leading to noticeable reductions in brain volume and alterations in synaptic and axonal damage. Another model that mimics chronic HIV-1 exposure was assessed by infecting human cell reconstituted SCID mice and monocyte-derived macrophages. This model utilized a macrophage-tropic HIV-1 molecular clone expressing either Tat 72 or Tat 101, and it was observed that the second exon of Tat was required for efficient replication within macrophages (Neuveut et al., 2003). While molecular alterations caused by genetic variation of Tat, specifically Tat length, have been characterized in *in vitro* cell culture systems, behavioral alterations have not been characterized. Various behavioral tests are available, such as: open field, elevated plus mazes, marble burying tests, acoustic startle response, and pre-pulse inhibition, which are used for determining anxiety-like responses and correlating body flinching to neuronal damage, respectively (Fitting et al., 2008; Paris et al., 2014, 2015). While behavior alterations have been examined in Tat 72 and 86 in mice and rats, to our knowledge, there have not been investigations to determine if different Tat lengths, specifically Tat 101, will cause detectable alterations in animal behavior.

As previously mentioned, Tat is synthesized by two exons, primarily resulting in a protein of 101 amino acids, however, early clinical isolates of subtype B HIV-1 Tat encoded for a 86 amino acid variant, which led to the prominent use of Tat 72 and 86 in a multitude of studies. Based on this literature meta-analysis, we were able to conclusively demonstrate that although the most biologically relevant, Tat 101 is not the most frequently used variant. There does not appear to be an increase in the number of publications that utilize the Tat 101 (Figure 1), however, as stated above, functional differences of Tat have been observed when using different length variants. Therefore, due to recent publications focused on subtype B Tat length (van der Kuyl et al., 2018; Link et al., 2019), and the functions that are associated with the second exon of Tat, it is the opinion of the authors that future studies utilize both Tat 86 and Tat 101 whenever possible, but if only one can be used to use Tat 101 to ensure biological relevance to the studies performed (Jeang et al., 1999; Marcello et al., 2001; López-Huertas et al., 2010; van der Kuyl et al., 2018; Link et al., 2019).

## Author Contributions

AM conceived the study. AM and MN designed the study. AM, WD, JM, BW, and MN prepared and designed the figures and drafted the manuscript. All authors have read and approved the final manuscript.

### Conflict of Interest

The authors declare that the research was conducted in the absence of any commercial or financial relationships that could be construed as a potential conflict of interest.

## Supplementary Material

Supplemental Table 1 Publication results from a PubMed search for HIV-1 Tat from 2009 to 2018. Reported here are the publications used for the analysis in Figure 1. The parameters used on the PubMed search were: (HIV-1 Tat) NOT “review”[Publication Type], 2009/01/01 to 2018/12/31, and free full text. 973 publications were examined for their utilization of Tat, whether they were: a recombinant protein, expressed within an animal model, derived from a plasmid, or used in a molecular simulation. Publications that did not explicitly state the length of Tat utilized or the molecular clone that it was derived from were listed as *Tat length not stated in publication* (*LNS*). The majority of HIV-1 transgenic animal models are Tat 86, however, if it was not explicitly stated it was reported as *LNS*. There were publications that were not accessible and those were listed as *No access to publication* (*NAP*). Additionally, publications that are listed as *Tat peptides* (*PEP*) refers to any truncations of Tat that are not 72 or 86 residues in length. Publications that solely examined the sequences of patients (i.e., Did not utilize a molecular clone that would express Tat, a recombinant protein, a transgenic animal model, or an expression vector) were listed as *No Tat used in this publication (NTU)* and not included in Figure 1 (*n* = 124). Publications that examined SIV rather than HIV were listed as *SIV* and were also not included in Figure 1 (*n* = 3). Abbreviation used in this document are: PEP, Tat peptide; 72, Tat 72; 86, Tat 86; 101, Tat 101; NTU, No Tat used in this publication; LNS, Tat length not stated in publication, and NAP, no access to publication.
